# DNA methylation age is associated with mortality in a longitudinal Danish twin study

**DOI:** 10.1111/acel.12421

**Published:** 2015-11-17

**Authors:** Lene Christiansen, Adam Lenart, Qihua Tan, James W. Vaupel, Abraham Aviv, Matt McGue, Kaare Christensen

**Affiliations:** ^1^ The Danish Aging Research Center, and The Danish twin RegistryInstitute of Public HealthUniversity of Southern DenmarkOdenseDenmark; ^2^ Max Planck Odense Center on the Biodemography of AgingInstitute of Public HealthUniversity of Southern DenmarkOdenseDenmark; ^3^ Department of Clinical GeneticsOdense University HospitalOdenseDenmark; ^4^ Max Planck Institute for Demographic ResearchRostockGermany; ^5^ The Center for Human Development and AgingNew Jersey Medical SchoolUniversity of Medicine and Dentistry of New JerseyNewarkNJUSA; ^6^ Department of PsychologyUniversity of MinnesotaMinneapolisMNUSA; ^7^ Department of Clinical Biochemistry and PharmacologyOdense University HospitalOdenseDenmark

**Keywords:** biological age, biomarker, DNA methylation, epigenetic clock, mortality, twins

## Abstract

An epigenetic profile defining the DNA methylation age (DNAm age) of an individual has been suggested to be a biomarker of aging, and thus possibly providing a tool for assessment of health and mortality. In this study, we estimated the DNAm age of 378 Danish twins, age 30–82 years, and furthermore included a 10‐year longitudinal study of the 86 oldest‐old twins (mean age of 86.1 at follow‐up), which subsequently were followed for mortality for 8 years. We found that the DNAm age is highly correlated with chronological age across all age groups (*r* = 0.97), but that the rate of change of DNAm age decreases with age. The results may in part be explained by selective mortality of those with a high DNAm age. This hypothesis was supported by a classical survival analysis showing a 35% (4–77%) increased mortality risk for each 5‐year increase in the DNAm age vs. chronological age. Furthermore, the intrapair twin analysis revealed a more‐than‐double mortality risk for the DNAm oldest twin compared to the co‐twin and a ‘dose–response pattern’ with the odds of dying first increasing 3.2 (1.05–10.1) times per 5‐year DNAm age difference within twin pairs, thus showing a stronger association of DNAm age with mortality in the oldest‐old when controlling for familial factors. In conclusion, our results support that DNAm age qualifies as a biomarker of aging.

## Introduction

A growing body of evidence documents that epigenetic phenomena, such as DNA methylation, are implicated in disease development and rate of aging (Maunakea *et al*., 2010; Huidobro *et al*., 2013; Murphy & Mill, 2014).

Due to the advent of array technologies, such as the Infinium HumanMethylation27 and HumanMethylation450 BeadChips, DNA methylation levels of CG dinucleotides (CpGs) across the genome are now easily accessible markers. Such array‐based studies of the genomewide methylation landscape in humans have demonstrated a changing pattern throughout the entire lifetime: changes that are influenced by various parameters such as the genetic background, environmental exposures, and simple epigenetic drift or loss of maintenance of the epigenetic marks (Alisch *et al*., 2012; Bell *et al*., 2012; Florath *et al*., 2014).

Several recent studies have made use of the age‐related changes in methylation profiles to construct DNA methylation signatures, a DNA methylation age (DNAm age) or ‘epigenetic clock’, with impressively high correlations with chronological age, of about 0.7 or greater. These indexes have been based on profiles from whole blood (Hannum *et al*., 2013; Weidner *et al*., 2014), saliva samples (Bocklandt *et al*., 2011), or samples across multiple tissues (Koch & Wagner, 2011; Horvath, 2013).

Considering that methylation profiles are modifiable by lifestyle and other environmental influences, it has been proposed that DNAm age is a biomarker of aging, that is, that DNAm age provides a better estimate of biological age than chronological age and is associated with current and future health and mortality (Hannum *et al*., 2013; Weidner *et al*., 2014). Along this line of thought, Marioni *et al*. (2015a) recently tested whether the deviation of DNAm age from chronological age, ∆Age, was associated with all‐cause mortality in four large study cohorts (mean ages 66–79 years) with mean time to death ranging from 7.2 to 10.5 years. In a meta‐analysis across the four cohorts, the authors found that a 5‐year increase in ∆Age was associated with a moderately increased mortality risk of 21% using the Hannum index and 11% using the Horvath index, independent of chronological age and other known risk factors (Marioni *et al*., 2015a).

In this study, we estimated DNAm age using the frequently applied Horvath prediction model and confirmed it using the Hannum prediction model. The study sample consisted of 378 twins aged 30–82 years from the Danish Twin Registry. The oldest 86 twins (mean age 76.2 years at intake) were resampled in a 10‐year follow‐up study and had methylation age determined again at mean age 86.1 years. The mortality in this sample was subsequently followed for 8 years. The twin design enabled us to control partly for genetic and rearing environment in the mortality study.

Based on these cross‐sectional and longitudinal data, we found that the association between DNAm age and chronological age diminished substantially with age. The finding may in part stem from selective mortality, as DNAm age was associated with mortality among the oldest‐old.

## Results

Table 1 outlines the basic characteristics of the study populations: the cross‐sectional data from young and middle‐aged twins drawn from the birthweight‐discordant twin study and the cross‐sectional and longitudinal data from the study of elderly twins. As the birthweight‐discordant study group is a selected sample, we tested for any linear or nonlinear relationship between birthweight and DNAm age and found no association (*P*'s > 0.25, data not shown).

**Table 1:** Characteristics of the study populations

Data	Sex	Age (SD)	Horvath methylation age (SD)	Age difference (SD)	*N*
Cross‐sectional (*N* = 292)	Male	47.82 (15.40)	48.76 (14.18)	0.95 (4.82)	152
	Female	49.45 (15.54)	49.31 (13.67)	−0.14 (5.41)	140
Young (*N* = 142)	Male	33.18 (1.86)	35.88 (3.84)	2.70 (3.79)	78
	Female	32.98 (2.17)	35.77 (4.87)	2.78 (3.98)	64
Middle‐aged (*N* = 150)	Male	63.24 (4.06)	62.34 (5.99)	−0.90 (5.10)	74
	Female	63.32 (4.18)	60.71 (6.17)	−2.61 (5.25)	76
Longitudinal (*N* = 172)	Male	81.70 (5.28)	77.94 (5.63)	−3.76 (4.63)	48
	Female	80.97 (5.27)	76.29 (5.86)	−4.68 (5.23)	124
Intake (*N* = 86)	Male	76.75 (1.73)	74.37 (3.40)	−2.39 (3.73)	24
	Female	76.02 (1.76)	72.65 (4.51)	−3.37 (4.88)	62
Follow‐up (*N* = 86)	Male	86.64 (1.74)	81.51 (4.70)	−5.13 (5.10)	24
	Female	85.92 (1.77)	79.94 (4.67)	−5.98 (5.29)	62

	Min	*Q*_1_	Mean	Median	*Q*_3_	Max
Follow‐up interval (years)	9.47	9.55	9.90	10.01	10.09	10.54
Methylation age difference (years)	0.51	5.14	7.25	6.93	9.26	17.56

**Figure 1:**
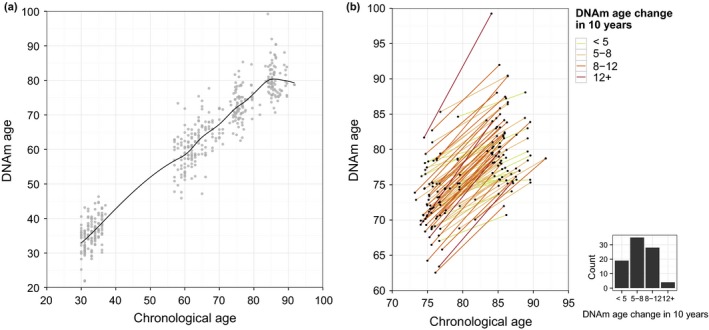
Horvath DNAm age against chronological age. (a) Correlation between the methylation age using the Horvath model. The relation is visualized by a smoothing spline. (b) DNAm age trajectories of each individual in the longitudinal study of oldest‐olds. The lines are colored by intervals of slow to fast agers, and the histogram indicates the distribution within the intervals.

In Fig. 1a, the estimated Horvath DNAm age is plotted against the chronological age for the three groups. The overall correlation coefficient, through all age groups, was 0.97. We fitted a cubic smoothing spline to visualize the relationship between DNAm age and chronological age. As Fig. 1a shows, at younger ages the DNAm age increases in line with the chronological age; however, at older ages the increase in DNAm with chronological age decelerates. The Horvath index predicted age slightly lower than chronological age (mean = 1.4 years lower, SD = 5.6). The Hannum index supported the general picture (results not shown), although these estimates were higher than chronological age through the entire age range (mean = 3.6 years higher, SD = 5.4).

To further explore the DNAm age deceleration, we separately regressed DNAm age onto chronological age for the young and middle‐aged cross‐sectional age groups as well as for the longitudinal sample of oldest‐olds (see Fig. S1, Supporting information). This analysis shows that DNAm age is closely related to actual age in the young population with DNAm age increasing 0.97 years (CI_95_: 0.65–1.29) for each year increment in chronological age. However, the average change of methylation age by actual age attenuates in the middle‐aged populations (0.80 years, CI_95_: 0.59–1.00), and even more so in the longitudinal analysis of the oldest population (0.65 years, CI_95_: 0.51–0.78).

In Fig. 1b, the trajectories for each longitudinally followed individual are plotted, with the individual lines grouped by color into four classes of DNAm age increase over the approximately 10‐year increase in chronological time, ranging from < 5 years (slow DNAm agers) (22%), over 5–8 years (41%) and 8–12 years (33%), to more than 12 years (faster DNAm agers) (5%).

The apparent decrease in rate of change of DNAm age with increasing age could be partly due to selective survival of those individuals that present with a DNAm age younger than chronological age. This hypothesis was supported by a Cox proportional hazard analysis of the second (follow‐up) assessment of the Horvath DNAm age. Of the 86 individuals, 55 had died during the mean mortality follow‐up time of 5.7 years (range 0.3–7.9), and the hazard ratio per 5‐year increase in DNAm age vs. chronological age was estimated to be 1.35 (CI_95_: 1.04–1.77, *P* = 0.019). The Hannum predictor gave a similar estimate, although nonsignificant (1.35, CI_95_: 0.94–1.95, *P* = 0.106).

**Figure 2:**
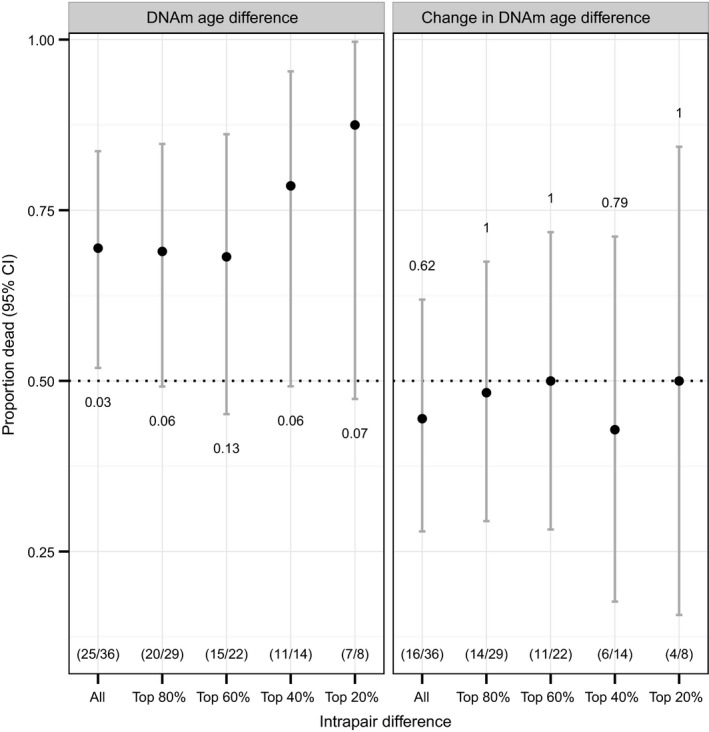
Mortality of the oldest twins according to intrapair DNAm age difference at follow‐up and difference in change over 10 years. The plots display the proportion of twins where the DNAm oldest twins died first (Proportion dead), for all twins and divided into groups with increasing intrapair difference. The numbers of twin pairs in each group are given in brackets, and the *P*‐values are shown below the lines on the left panel and above the lines on the right.

To control partly for genetic and environmental factors shared between a twin pair, we performed intrapair mortality analysis of the old twins from the second assessment onwards for 8 years. The two plots in Fig. 2 depict the mortality according to intrapair DNAm age difference as assessed by the start of the mortality follow‐up time on the one side, and difference in change over the preceding 10 years on the other side. At the end of the follow‐up, 36 of the 43 twin pairs had at least one deceased twin, and in these 36 pairs, the twin partner with the higher DNAm age died first in 25 cases (Probability = 0.69, CI_95_: 0.52–0.84) corresponding to a more‐than‐double risk of dying first for the DNAm oldest co‐twin. The probability increased with increasing discordance in DNAm age within twin pairs in the cross‐sectional estimation, whereas the size of the intrapair difference in change of methylation age vs. actual age over the 10 preceding years did not affect the probability of dying first (Fig. 2). In addition, a logistic regression analysis showed that while accounting for cell composition of the 36 twin pairs, the odds of dying first increased 3.2 (1.05–10.1) times for a 5‐year DNAm age difference within twin pairs. The corresponding estimate using the Hannum model was 2.1 (0.9–4.8).

## Discussion

Using a sample of 378 twins, age 30–82 years, we confirm the previous findings that DNAm age is correlated well with chronological age in young and middle‐aged individuals with a tendency of an underestimation with increasing age. This finding is supported by data from Marioni *et al*. (2015b) who find that the Horvath clock underestimates the age in older individuals by approximately 4 years.

The lower yearly increase in DNAm age with increasing age may be partly explained by selective mortality; that is, those with high DNAm have the highest mortality, and hence, the surviving population will increasingly comprise slow agers with low methylation ages making the DNAm age curve level off when plotted against chronological age in a cross‐sectional study (Christensen *et al*., 2008; Vaupel, 2010). The less‐than‐perfect relationship in the longitudinal study of oldest‐olds also suggests that methylation age is advancing at a slower rate than chronological age. In principle, it is possible that this phenomenon is related to an evolution of hematopoietic oligoclonality at extreme ages, which was recently suggested (Holstege *et al*., 2014), where the peripheral blood cells in long‐living individuals are derived mainly from clones with a younger DNAm age.

In the mortality analysis, we confirmed the hypothesis of selective mortality in a classical survival analysis showing that a 5‐year higher DNAm age than chronological age was significantly associated with a 35% increase in mortality risk. Due to the twin setup of this study, we were further able to substantiate this with an intrapair twin analysis, which demonstrated that among these old twins, the twin partner with the higher DNAm age in 69% of cases were the twin that died first, which corresponds to a more‐than‐double risk for the DNAm oldest twin compared to the co‐twin. Moreover, we saw a clear tendency of a dose‐effect of accelerated methylation age, as the larger the intrapair difference in DNAm age, the higher the probability of mortality in the DNAm oldest twin. These findings thus support that DNAm age is a useful and powerful biomarker of aging.

Our findings are in line with the only other study thus far that has explored the possible association between DNAm age and mortality (Marioni *et al*., 2015a), although the effect estimates were stronger (35% vs. 11% for the Horvath clock and 21% for the Hannum clock, respectively). With a combined sample size of more than 4000 individuals, from four independent cohorts of older people, and with a much higher number of deaths (> 800), the study from Marioni *et al*. was considerably larger than ours. However, the use of the powerful twin design in the present study may explain the relatively stronger association, which is likely to be due to the elimination of genetic and early life confounding in the intrapair analysis. Individuals may already very early in life have a DNAm age that deviates from the mean due to genetic factors and/or rearing environment, and the intrapair analyses will to some degree control for such early‐life differences.

The combined results from the mortality studies support the potential of blood methylation age as a biomarker of future general health and all‐cause mortality. In addition, the ability of the DNAm age index to discriminate between slowed and accelerated rate of aging holds potential for identifying genes, pathways, and regulatory mechanisms of importance for disease development and age‐related decline in biological functioning. To this end, studies are now emerging which demonstrate that an accelerated DNAm age may be linked to disease development. For instance, a modest cross‐sectional association between accelerated methylation age and lower cognition, weaker grip strength, and poorer lung function was suggested by Marioni *et al*. (2015b), although they could not document that DNAm age at intake could predict future decline in any of the measured parameters. In addition, a significantly accelerated DNAm age of both blood and brain tissue was seen in trisomy 21 individuals, perhaps mirroring the premature aging conditions often experienced by these individuals (Horvath *et al*., 2015a). Further exploring the possible importance of tissue specific aging, obesity has been associated with an increased biological age of the liver (Horvath *et al*., 2014). And recently, Horvath *et al*. (2015b) applied the ‘epigenetic clock’ to a number of tissues and found that the cerebellum ages at an increasingly slower rate in 80+ years individuals compared to other brain regions. While the conclusions that can be drawn from this finding are still highly speculative, it does suggest that DNAm age of also specific tissues could be a sign of biological age (Horvath *et al*., 2015b).

In conclusion, we report a leveling off of the correlation between DNAm age and chronological age in old cohorts, which is likely to reflect that individuals reaching advanced ages have a more healthy aging profile. The association with mortality risk seen in the old twins when adjusting for familial factors adds considerable evidence to support the hypothesis that DNAm age can discriminate between slow and fast agers, consistent with the hypothesis that DNAm age is a proxy for underlying mechanisms of aging.

## Experimental procedures

### Study samples

The study samples were drawn from two surveys conducted at the Danish Twin Registry (Skytthe *et al*., 2013) for which data on DNA methylation were already available: the Longitudinal Study of Aging Danish Twins (LSADT) (McGue & Christensen, 2007) and a study of the Extremely Birth Weight Discordant Twins (Frost *et al*., 2012).

The Longitudinal Study of Aging Danish Twins includes all Danish twins 70 years of age or more. The study was initiated in 1995 and surviving twins were followed‐up every second year through 2007 (Christensen *et al*., 1999; McGue & Christensen, 2007). In 1997 and 2007, whole blood samples were collected from same‐sex twin pairs, and the samples included in the present study consisted of those 18 monozygotic (MZ) pairs and 25 dizygotic (DZ) pairs, which participated in both the 1997 and the 2007 wave. The age range for LSADT samples was 73–82 years (mean 76.2) in 1997.

The second sample consisted of 146 birthweight‐discordant MZ twin pairs collected in 2008–2010 (Frost *et al*., 2012). The sample was divided into two age groups, with 142 participants aged 30–37 (mean 33.1) years and 150 participants aged 57–74 (mean 63.3) years.

Zygosity determination of all twin pairs was based on 12 highly polymorphic microsatellite markers.

### DNA methylation data

DNA was isolated from buffy coats using the salt precipitation method applying either a manual protocol (Miller *et al*., 1988) or a semi‐automated protocol based on the Autopure System (Qiagen, Hilden, Germany). Bisulfite treatment of 500 ng genomic DNA was carried out with the EZ‐96 DNA methylation kit (Zymo Research, Orange County, CA, USA) following the manufacturer's protocol. Genomewide DNA methylation was measured using the Infinium HumanMethylation450K BeadChip (Illumina, San Diego, CA, USA) at the Leiden University Medical Center and in accordance with the manufacturer's instructions. Data preprocessing was performed using the free r package *minfi* (Maksimovic *et al*., 2012), and a DNA methylation level was summarized for each CpG site by calculating a ‘*beta*’ value ranging from 0 to 100%. CpG probes were treated as missing if the detection *P*‐value > 0.01, and CpG sites with more than 5% missing data were excluded from the analysis.

DNA methylation data have been deposited in the NCBI GEO database (http://www.ncbi.nlm.nih.gov/geo/) under accession numbers GSE61496 and GSE73115.

### Statistical analysis

For each twin, we calculated the DNAm age following the protocol of Horvath (2013) and Hannum *et al*. (2013). In the case of both the birthweight‐discordant twin sample and the LSADT sample, we visualized the association between chronological age and DNAm age by fitting a cubic smoothing spline and further estimated correlations by linear regression.

In the case of the birthweight‐discordant twin sample, we additionally tested the relationship between birthweight and DNAm age using a linear mixed regression model with birthweight, age, and sex as fixed effects and twin pairing as a random factor. A nonlinear relationship between birthweight and DNAm age was assessed by converting birthweight into dummy variables according to its 0.25, 0.5, and 0.75 quantiles and using the first group as reference.

In the case of the LSADT, we estimated the population average (marginal) and within‐twin pair (conditional) association of DNAm age with mortality. The twins included in the mortality study all participated in the 10‐year follow‐up, and therefore, the mortality assessment started after the follow‐up sample was drawn. In all survival analyses, the possible effect of cell composition was estimated and adjusted for by the approach proposed by Houseman *et al*. (2012). To assess the conditional influence of estimated methylation age, first, we computed the proportion of the co‐twins with a higher DNAm age who died first and compared it with the null hypothesis that both co‐twins are equally likely to die first by an exact binomial test. Second, we used a conditional logistic regression stratified by twin pairs and adjusted for cell composition to measure the influence of within‐twin pair DNAm age difference on the odds of dying first. To measure the marginal effect of DNAm age on survival, a Cox proportional hazards regression stratified by sex and adjusted for cell composition was employed. As some twins might have died before they could have been included in the study, all co‐twins in the study were considered left truncated at the age of entering the follow‐up. The Cox regression was performed using a robust sandwich estimator of standard errors to account for correlation between twin pairs. Schoenfeld residuals were checked for deviations from the proportional hazards assumption. All statistical analyses were carried out using r 3.1.3 (R Core Team 2015 ‐ https://www.r-project.org).

## Funding

This work was supported by grants from the European Union's Seventh Framework Programme (FP7/2007‐2011) under grant agreement no. 259679, and The Danish National Program for Research Infrastructure 2007 [09‐063256].

## Conflict of interest

The authors declare no conflict of interest.

## Author contribution

Lene Christiansen was involved in conception and design of the study, acquisition of data, interpretation of data, and drafting the manuscript. Adam Lenart was involved in analysis of data, interpretation of data, critical revision of the manuscript, and approval of the final version of the manuscript. Qihua Tan was involved in analysis of data, interpretation of data, critical revision of the manuscript, and approval of the final version of the manuscript. James Vaupel was involved in conception and design of the study, critical revision of the manuscript, and approval of the final version of the manuscript. Abraham Aviv was involved in conception and design of the study, critical revision of the manuscript, and approval of the final version of the manuscript. Matt McGue was involved in conception and design of the study, critical revision of the manuscript, and approval of the final version of the manuscript. Kaare Christensen was involved in conception and design of the study, acquisition of data, interpretation of data, and drafting the manuscript.

## Supporting information


**Fig. S1** Horvath DNAm age against chronological age separated into young, middle‐aged, and old cohorts.
