# Supplementary material for: DNA methylation age is associated with mortality in a longitudinal Danish twin study
Source: Aging Cell. 2015 Nov 17;15(1):149–54. doi: 10.1111/acel.12421 (PMC4717264; doi:10.1111/acel.12421)
Supplement: Supplementary file 1 — Fig. S1 Horvath DNAm age against chronological age separated into young, middle‐aged, and old cohorts. [file ACEL-15-149-s001.pdf]

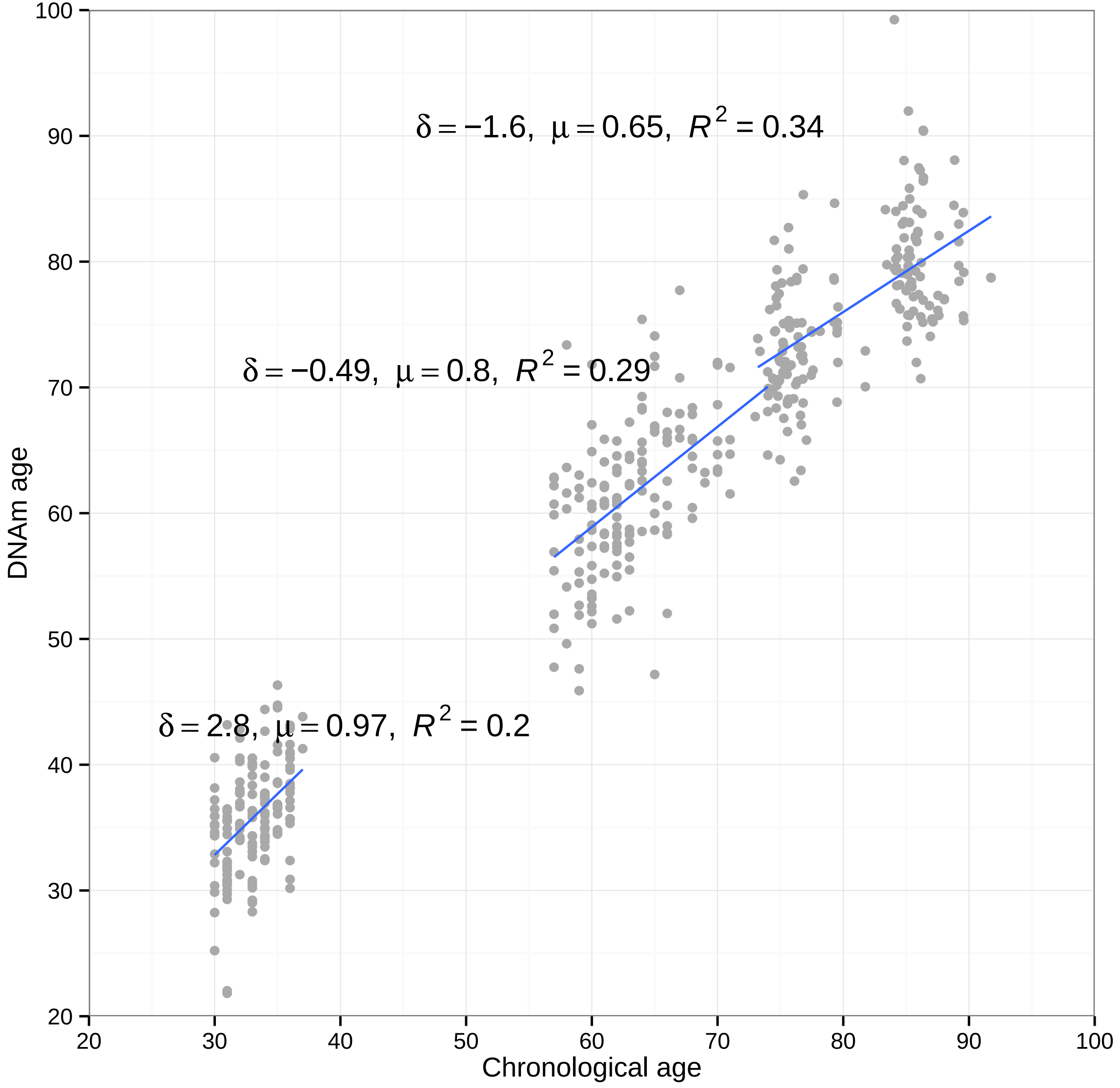

δ = −1.6, μ = 0.65, R^2 = 0.34
δ = −0.49, μ = 0.8, R^2 = 0.29
δ = 2.8, μ = 0.97, R^2 = 0.2
DNAm age
Chronological age
20
30
40
50
60
70
80
90
100

Figure S1: Horvath's DNAm age against chronological age separated into young, middle-aged and old cohorts.

We separately regressed DNAm age onto chronological age for the young and middle-aged cross-sectional age groups as well as for the longitudinal sample of oldest-olds to estimate two parameters: δ, or intercept, which gives the average difference between DNAm and chronological age, and so indexes whether there is systematic over- or under-prediction of age. Further, μ or slope gives the expected increase in DNAm age for every year increase in chronological age. R^2 denotes the square of the correlation for the sub-groups.
